# Elevated CO_2_ can modify the response to a water status gradient in a steppe grass: from cell organelles to photosynthetic capacity to plant growth

**DOI:** 10.1186/s12870-016-0846-9

**Published:** 2016-07-12

**Authors:** Yanling Jiang, Zhenzhu Xu, Guangsheng Zhou, Tao Liu

**Affiliations:** State Key Laboratory of Vegetation and Environmental Change, Institute of Botany, Chinese Academy of Sciences, 20 Nanxincun, Xiangshan, Haidian Beijing, 100093 China; Chinese Academy of Meteorological Sciences, China Meteorological Administration, 46 Zhongguancun South Street, Haidian Beijing, 100081 China

**Keywords:** Elevated CO_2_, Grassland, Mesophyll cell organelle, Photosynthetic capacity, Plant growth analysis, Water status gradient

## Abstract

**Background:**

The atmospheric CO_2_ concentration is rising continuously, and abnormal precipitation may occur more frequently in the future. Although the effects of elevated CO_2_ and drought on plants have been well reported individually, little is known about their interaction, particularly over a water status gradient. Here, we aimed to characterize the effects of elevated CO_2_ and a water status gradient on the growth, photosynthetic capacity, and mesophyll cell ultrastructure of a dominant grass from a degraded grassland.

**Results:**

Elevated CO_2_ stimulated plant biomass to a greater extent under moderate changes in water status than under either extreme drought or over-watering conditions. Photosynthetic capacity and stomatal conductance were also enhanced by elevated CO_2_ under moderate drought, but inhibited with over-watering. Severe drought distorted mesophyll cell organelles, but CO_2_ enrichment partly alleviated this effect. Intrinsic water use efficiency (WUE_i_) and total biomass water use efficiency (WUE_t_) were increased by elevated CO_2_, regardless of water status. Plant structural traits were also found to be tightly associated with photosynthetic potentials.

**Conclusion:**

The results indicated that CO_2_ enrichment alleviated severe and moderate drought stress, and highlighted that CO_2_ fertilization’s dependency on water status should be considered when projecting key species’ responses to climate change in dry ecosystems.

**Electronic supplementary material:**

The online version of this article (doi:10.1186/s12870-016-0846-9) contains supplementary material, which is available to authorized users.

## Background

The IPCC’s Fifth Assessment Report (AR5) showed that atmospheric carbon dioxide concentration had increased by 40 % since the pre-industrial era, reaching ~390 μmol mol^−1^ in 2011. It is predicted to rise to 500 μmol mol^−1^, perhaps even above 900 μmol mol^−1^, by the end of this century. Continued emissions of greenhouse gases will cause further warming and precipitation changes [1]. The effects of elevated CO_2_ concentration (elevated CO_2_) and climatic change on plants—from the molecular basis to physiological processes, individual growth, and vegetation productivity aspects—have attracted considerable attention for several decades (e.g., [2–6]).

Many studies have reported the biological responses of plants to CO_2_ enrichment and its interactions with other environmental factors (e.g., [3, 4, 6]). The effects of elevated CO_2_ include enhanced net photosynthesis rate (*A*_net_), down-regulated stomatal conductance (*g*_s_) [3, 7–9], dilution of chemical elements [10], imbalance of sink–source relationships [11, 12], increased plant growth and vegetation productivity [2, 13], changes in species competition interactions and community structure [13–15], and lengthened growing seasons [16]. However, these elevated CO_2_-induced changes might be mediated by other environmental factors, particularly changes of water availabilities. Severe drought adversely affects plant growth, gas exchange, and photosynthetic activity [17], but elevated CO_2_ might partly alleviate the harmful impact of water deficit stress on these biological processes, and even survival [18–21]. Elevated CO_2_ can enhance plant resistance to water deficit stress by mitigating oxidative damage, maintaining *A*_net_ and decreasing *g*_s_, and improving the plant water status, thereby raising the water use efficiency (WUE) [4, 21–24]. Under moderate water stress, a marked stimulation occurs due to elevated CO_2_ [22–26]. However, over-watering can reduce, or even reverse this stimulation [27–30]. Nevertheless, few studies on the combined effects of elevated CO_2_ and a water status gradient have been conducted, particularly on multiple scales.

*Agropyron cristatum* (L.) Gaertn, or crested wheatgrass, a C_3_ species, is a dominant species in the steppe regions of Central Asia, and is also widespread in western North America [31, 32]. It is a perennial herb native to North China with good palatability and high forage value. Moreover, its prosperity is recognized as an indicator of degradation of the steppe ecosystem in the context of overgrazing and adverse climate change. For instance, degradation might initially occur if crested wheatgrass thrives relative to other dominant species such as *Leymus chinensis* and *Stipa grandis* [33]. Thus, this species is crucial for assessing the vulnerability and restorative capacity of the semiarid region, as well as forage resource management. These temperate grasslands, which are dominated by several major grasses including *A. cristatum*, have been severely degraded during recent decades because of adverse climatic change and improper land use [34–37]. Although this arid region is projected to become drier and hotter, excessive precipitation events may occur more frequently [38, 39]. This would further threaten the ecosystem function including dominant species growth and survival [36, 37, 40]. The leaf-level instantaneous responses of *A*_net_, *g*_s_, and WUE to elevated CO_2_ have been quite well investigated [4, 6, 8]. To our knowledge, however, few prior efforts have been made to examine the effects of elevated CO_2_ under a wide-ranging water status gradient (seven watering treatments from extremely severe deficit to relative over-watering) (but see Manea and Leishman, [15]), particularly integrating multiple variables from organelle structure to physiological processes, individual morphology and structure, biomass allocation, and plant growth aspects. In this experiment, structural and physiological traits were examined to find sensitive indicators, and to summarize the adaptive strategy of *A. cristatum* to climatic changes. The objective of the current study was to test the hypotheses: (1) elevated CO_2_ modifies the effects of soil water status on the dominant species, with stimulation in a moderate range of water status changes, no positive response with over-watering, but an alleviation of damage from severe water deficit; and (2) associated responses co-occur at the mesophyll cell ultrastructure, photosynthetic physiological activity, plant growth and structure levels under elevated CO_2_ and different water conditions.

## Methods

### Plant culture

Each PVC plastic pot (9.7 cm in diameter, 9.5 cm in height, 0.70 L) was filled with 0.68 ± 0.01 (±SE, *n* = 60) kg of dry soil, and planted with four plants per pot. The soil was retrieved from the local soil surface (0–30 cm), and *A. cristatum* seeds were collected the year before the experiment from the local steppe—a typical grassland ecosystem in Xilinhot (43°38′N, 116°42′E, 1100 m a.s.l.), Inner Mongolia, China. The soil was a castanozem type, with a medium texture containing 29.0 %, 31.2 %, and 39.8 % clay (<5 μm particle diameter), silt (5–50 μm), and sand (>50 μm), respectively. The soil organic carbon, total nitrogen, and available nitrogen concentrations were 12.31 ± 0.19 g kg^−1^, 1.45 ± 0.02 g kg^−1^, and 81.61 ± 0.71 mg kg^−1^, respectively. The soil water field capacity (FC) and permanent wilting point were 25.8 and 6.0 % (w/w), respectively, and the gravimetric bulk density was 1.21 g cm^−3^. With a mean annual temperature of 2.95 °C and mean annual precipitation (MAP) of 266 mm over the past 30 years, this region is characterized by a continental temperate climate with a dry, frigid winter (−18.6 °C lowest mean month temperature in Jan), and a wet, hot summer (21.4 °C maximal mean month temperature in Jul). Most precipitation (88 %) occurs in the growth season from May to Sep. To obtain uniform seedlings [26, 41], all pots were initially placed in a naturally illuminated glasshouse (day/night temperatures of 26–28/18–20 °C) until the third leaf emerged (27 days after sowing), and then transferred into two open-top chambers (OTCs) to subject the plants to two-month watering and increased CO_2_ concentration treatments. Water was provided by a sprayer at around 17:00 every 3 days according to previous similar experiments [26, 35].

The OTCs were established in a regular hexahedron shape (each side 85 cm wide, 150 cm high) with a top-opening rain shelter on the top and a space below for free exchange of gases between the OTC and ambient atmosphere. Pure CO_2_ gas (99.999 %) was supplied from a cylinder (Chao Hong Ping Gas Co. Ltd, Beijing, China), and a CO_2_ gas sensor (eSENSE-D, SenseAir, Delsbo, Sweden) was used to continuously monitor and control the CO_2_ concentration over 24 h with a data logger (DAM-3058RTU, Altai Sci Tech Dev Co. Ltd., Shang Hai, China). The CO_2_ concentrations had a ±30 μmol mol^−1^ change relative to the set points. The air temperature and relative humidity (RH) were monitored using thermocouples (HOBO S-TMB-M006, Onset Computer Co., Bourne, MA, USA) and humidity transducers (HOBO S-SMA-M005) installed at 75 cm height in and out of the chambers, respectively. Climatic data were automatically recorded and collected by a logger (HOBO H21-002) every 30 min during the experiments; day/night temperatures were 28.3 ± 5.7/22.7 ± 4.2 °C and RH was 62.4 ± 4.0 % in the OTCs, which were 1.3/0.5 °C greater and 3.5 ± 2.3 % lower than outside the OTCs. The OTC system has been proven to have acceptable effectiveness, relative availability, and data comparability for assessing the effects of elevated CO_2_ with climatic change at low cost [42, 43], although data interpretation needs to be cautious in various environmental contexts [44, 45].

### Experimental design

The experiment was designed with two factors, two CO_2_ concentrations (ambient CO_2_, ~390; elevated CO_2_, 550 μmol mol^−1^) and a seven-level precipitation gradient: W_−60_ (−60 % relative to mean precipitation in the local site over 30 years, as an extreme drought treatment), W_−30_ (−30 % relative to local mean precipitation), W_−15_ (−15 % relative to local mean precipitation), W_0_ (local mean precipitation, the control watering treatment), W_15_ (+15 % relative to local mean precipitation), W_30_ (+30 % relative to local mean precipitation, an over-watering treatment relative to the normal local precipitation), and W_60_ (+60 % relative to local mean precipitation), roughly equaling 147.0, 257.3, 312.5, 367.6, 422.7, 477.9, and 588.1 mm of precipitation during the growth season, respectively. Two OTCs were used, with separate irrigation treatments within each one as a split plot and 3–5 replicates (pots/treatment). A total of 80 plots were included initially; some pots were kept in reserve in case of experimental or plant growth problems. Plants were randomly placed within each OTC initially, replaced every 3 days, and transferred between the two chambers weekly (CO_2_ target points were switched simultaneously) to minimize any differences between growth chambers except for the desired treatments—CO_2_ concentration and water status [26, 46, 47].

### Soil water status and water use

The soil water content (SWC, g water g^−1^ dry soil) during the experiment was determined by weighing pots. The soil dry weight (SDW) at sowing was calculated as (TW − PW) × (1- SWC_0_) at sowing (the SWC_0_ was determined before sowing by oven-drying soil samples; there was no water drainage because the pots used had no holes, and plant weight was neglected). The SWC during the experiment was expressed as (TW − SDW − PW)/SDW, and the soil relative water content (SRWC) was expressed as SWC/FC × 100. Water use, i.e., actual evapotranspiration during the entire experiment, was derived from a water balance equation; water use = TW at harvest − TW at initial time + applied water amount. Thus, total biomass water use efficiency (WUE_t_) could be estimated as total plant biomass/water use [48]. TW is the total weight of the pot plus soil at each measurement time; PW is the net pot weight determined before filling with soil; FC is the SWC measured 24 h after fully wetting the soil.

### Plant biomass and leaf area

Each plant was separated into four parts, the stem, root, green leaves, and dead leaves, at both the start and end of the experiment, dried at 75 °C to a constant weight, and then weighed to get the biomass. Before drying, plant height, tiller and green leaf numbers were recorded; and green leaf area per plant, and the leaf parts used to determine gas exchange parameters were measured with a WinFOLIA system for root/leaf analysis (WinRhizo, Régent Instruments, Quebec, Canada).

### Plant growth analysis

Plant growth analysis was performed following Poorter [49]. The relative growth rate of each individual (RGR, mg g^−1^ day^−1^) was expressed as (ln *w*_2_ − ln *w*_1_)/(*t*_2_ − *t*_1_), where *w*_2_ and *w*_1_ are the biomass at final and initial harvest dates, respectively, and *t*_2_ and *t*_1_ indicate the two harvest times. The leaf mass ratio of total plant mass (LMR), stem mass ratio (SMR), and root mass ratio (RMR), as biomass allocation indicators, were expressed as percentages of leaf, stem, and root mass in the total plant mass, respectively. The plant/leaf morphological and structural indicators leaf area ratio (LAR, m^2^ kg^−1^; an indicator of the canopy size), leaf area and root mass ratio (LARMR, m^2^ kg^−1^; a proxy of the biomass balance between light-intercepting organs and resource element uptake organs), specific stem length (SSL, cm mg^−1^; a marker of the investment of stem carbon into plant height), and specific leaf area (SLA, m^2^ kg^−1^; an indicator of leaf thickness and compactness) were expressed as ratios of leaf area to whole plant mass, leaf area to root mass, stem length to mass, and leaf area to mass, respectively [49].

### Photosynthesis and chlorophyll *a* fluorescence

Leaf gas exchange and chlorophyll fluorescence were measured simultaneously using an open gas exchange system (LI-6400 F, LI-COR, Inc., Lincoln, NE, USA) combined with a leaf chamber fluorometer (LI-6400-40, LI-COR). Illumination was supplied to the leaves from a red-blue LED light source and the data were initially analyzed with data acquisition software (OPEN 6.1.4, LI-COR). Before measurement, the leaves were acclimated in the chamber for at least 10 min at 26–28 °C with a CO_2_ concentration of 390 μmol mol^−1^ and a photosynthetic photon flux density of 1500 μmol m^−2^ s^−1^, under which photosynthesis is nearly saturated, to obtain gas exchange parameters such as net light-saturated maximum photosynthetic rate (*A*_sat_), *g*_s_, transpiration rate (*E*), and intrinsic water use efficiency (WUE_i_, *A*_sat_/*E*). We measured at least three each of the youngest and fully expanded leaves from different individuals (one plant per pot) for all replicates, from 9:00 to 16:30 h daily. The vapor pressure deficit (VPD) in the cuvette was maintained at 1.7–2.7 kPa (2.39 ± 0.04, *n* = 492), possibly reflecting the actual conditions within the OTCs [26, 50]. The steady-state value of fluorescence (*F*_s_) was determined, and a second saturating pulse at 8000 μmol m^−2^ s^−1^ was imposed to determine the maximal light-adapted fluorescence (*F'*_m_). The actinic light was removed and the minimal fluorescence in the light-adapted state (*F'*_0_) was determined after 3 s of far-red illumination. The maximum photochemical efficiency of photosystem II (*F*_v_/*F*_m_) was determined midnight–predawn in completely dark-adapted leaves with a leaf fluorometer (LI-6400-40) linked to a LI-6400 F gas exchange system. The minimal fluorescence yield (*F*_0_) was determined under low modulated light of 1.0 μmol m^−2^ s^−1^, and the maximal fluorescence yield (*F*_m_) was obtained with a 0.8 s saturating pulse at ~8000 μmol m^−2^ s^−1^. The fluorescence parameters were calculated from the following formulae [51, 52]; the maximal efficiency of photosystem II (PSII) photochemistry is *F*_v_/*F*_m_ = (*F*_m_ − *F*_0_)/*F*_m_, and the efficiency of excitation energy captured by open PSII reaction centers is *F'*_v_/*F'*_m_ = (*F'*_m_ − *F'*_0_)/*F'*_m_.

### Estimation of *A*/*C*_i_ response curves

To analyze the *A*/*C*_i_ response curve to obtain key photosynthetic capacity parameters, a stepwise CO_2_ concentration gradient was implemented (390, 300, 200, 100, 50, 390, 390, 550, 800, and 1000 μmol m^−2^ s^−1^). Note, the third 390 value is not an error, but a trick to easily recover the ambient CO_2_ level from the lowest point of 50 μmol m^−2^ s^−1^. At each CO_2_ level, the leaves needed 2–3 min to equilibrate, and a match was also run to balance the CO_2_ and water vapor concentrations between the reference and leaf chambers. Furthermore, to estimate maximum rate of carboxylation (*V*_c,max_), maximum rate of electron transport (*J*_max_), and rate of thiose phosphate utilization (TPU), a curve-fitting software tool by Sharkey et al. [53] based on the method of Farquhar et al. [54] was run to analyze the *A*/*C*_i_ response data.

### Electron microscopy

For transmission electron microscopy, 2-mm^2^ pieces from the middle sections of the youngest and fully expanded leaves were dissected and immediately fixed in 2.5 % (v/v) glutaraldehyde in 0.1 M phosphate buffer (pH 7.0) overnight at 4 °C. The samples were then washed three times with the same buffer and post-fixed in 1 % osmium tetroxide overnight at 4 °C. After being washed in the same buffer, the leaf tissues were passed through an ethanol dehydration series, and then infiltrated and embedded in Spurr’s resin (Agar Scientific, Essex, UK). Sections were cut using an Ultracut R ultramicrotome (Leica, Wetzlar, Germany). The thin sections were stained with 2 % uranyl acetate and lead citrate [55], and then observed and photographed under a transmission electron microscope (JEM-1230, JEOL Ltd, Tokyo, Japan). For each treatment, three leaf samples were examined, and approximately 130 mesophyll cells were randomly chosen for the observations.

### Statistical analyses

A principal component analysis (PCA) was first conducted to test the relationships among the traits including the plant growth, structural, morphological, biomass allocation, and photosynthetic parameters, and the multivariate patterns of the effects of CO_2_ concentration and watering treatments alone and in combination [26, 56, 57]. Thereafter, we conducted an analysis of variance (ANOVA) for the plant growth, structural, morphological, biomass allocation, and photosynthetic traits, and the anatomical changes in mesophyll cells and their organelles with GLM Full Factorial Mode to test the main effects of watering, elevated CO_2_, and their interactions. Where watering treatments had a significant effect based on ANOVA, a multiple comparison was done with Duncan’s multiple range test. A one-way ANOVA was also conducted to test the differences between the two CO_2_ levels within the same watering treatment. The multiple factorial ANOVA model can be used for unequal variances and data near a normal distribution. All statistical analyses were made using the SPSS 20.0 software (SPSS Inc., Chicago, IL, USA). Unless otherwise noted, *P* < 0.05 was considered statistically significant.

## Results

### Soil water status

The watering treatments produced a wide-ranging soil water gradient combined with either ambient or elevated CO_2_ during the entire experimental period; when measured before watering at 17:00 every 3 days (the irrigation day) during a consecutive 10-day period, the SRWCs were 26.7 %, 39.4 %, 42.7 %, 45.6 %, 50.8 %, 53.3 %, and 61.7 % in the W_−60_, W_−30_, W_−15_, W_0_, W_+15_, W_+30_, W_+60_ watering treatments at ambient CO_2_, and 25.4 %, 36.1 %, 38.4 %, 44.0 %, 45.5 %, 50.9 %, and 51.5 % at elevated CO_2_, respectively (Additional file 1: Figure S1), indicating that elevated CO_2_ reduced the SRWC under every watering treatment (on average from 45.7 to 41.7 %, decreasing by 8.9 %). Watering and elevated CO_2_ exerted significant effects almost every day over the continuous 10-day period except for elevated CO_2_ concentration on the 57th and 62nd days after sowing according to a GLM ANOVA (Additional file 2: Table S1).

### Multiple plant traits and environmental effects

**Fig. 1:**
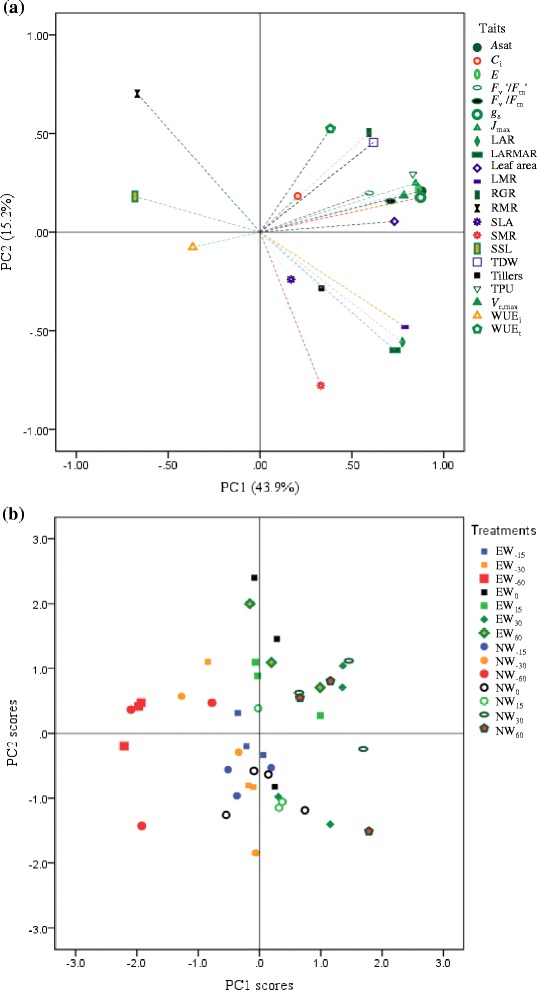
Principal component analysis on plant functional traits of *Agropyron cristatum* under elevated CO_2_ with water status gradient. The first two principal components (PCs) were shown (**a**), and projections of the two PCs were sorted by the combined treatments (**b**). *A*_sat_, light-saturated maximum photosynthetic rate; *C*_i_, intercellular CO_2_ concentration; *E*, transpiration rate; *g*_s_, stomatal conductance; *V*_c,max_, maximum rate of carboxylation; *J*_max_, maximum rate of electron transport; TPU, rate of thiose phosphate utilization; *F*_v_ *'*/*F*_m_ *'*, photochemical efficiency of open reaction centers of photosystem (PS) II; *F*_v_/*F*_m_, maximal photochemistry efficiency; LAR, leaf area ratio; LARMR, leaf area and root mass ratio; LMR, leaf mass ratio; RGR, relative growth rate; RMR, root mass ratio; SLA, specific leaf area; SMR, stem mass ratio; SSL, specific stem length; TDW, total plant biomass weight; WUE_i_, intrinsic water use efficiency; WUE_t_, total biomass water use efficiency. NW_−60_, NW_−30_, NW_−15_, NW_0_, NW_15_, NW_30_, NW_60_ denote −60 %, −30 %, −15 %, 0 %, 15 %, 30 %, and 60 % of watering relative to mean precipitation in the local site over 30 years with normal/ambient CO_2_ concentration; while EW_−60_, EW_−30_, EW_-15,_ EW_0_, EW_15_, EW_30_, EW_60_ are those with elevated CO_2_

PCA on multiple traits showed that the first and second principal components (PCs) explained 43.9 % and 15.2 % of the total variance, respectively (Fig. 1a). Most of the traits related to plant growth and photosynthetic activities were closely and positively correlated with PC1, and their loadings were mostly located in quadrant I. Those related to canopy size had similar correlations with PC1, and were mostly located in quadrant IV. Root and shoot biomass allocation traits were closely correlated with PC2, but separated conversely in quadrants II and IV, respectively. Projections for the multivariate traits and the effects of the two factors showed a complex pattern (Fig. 1b). However, the projections with extreme water deficit, including W_−60_ with ambient CO_2_ (NW_−60_) and W_−60_ with elevated CO_2_ (EW_−60_), and severe water deficit (NW_−30_, and EW_−30_) showed a distinct pattern, mostly appearing in the left part relative to the vertical line of origin, being opposed to those under relatively ample water conditions.

### Plant growth analysis

**Fig. 2:**
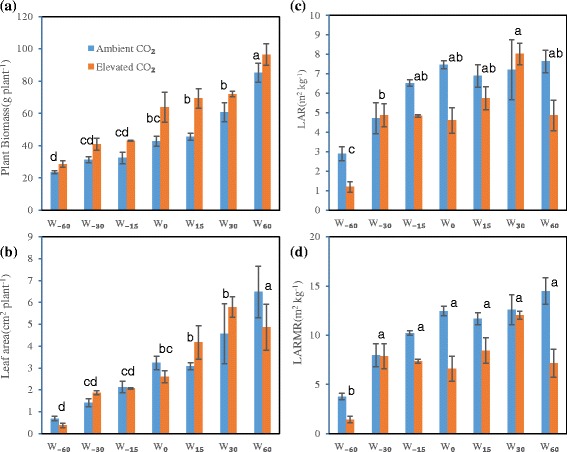
Effects of elevated CO_2_ on plant growth and its structure with water status gradient. **a** plant biomass; **b** leaf area; **c** LAR, leaf area and plant mass ratio; **d** LARMR, leaf area and root mass ratio. W_−60_, W_−30_, W_−15_, W_0_, W_15_, W_30_, and W_60_ represent −60 %, −30 %, −15 %, 0 %, 15 %, 30 %, and 60 % of watering relative to mean precipitation in the local site over 30 years. A GLM ANOVA between temperature and watering, and their interaction was show in Additional file 3: Table S2. Different lower case letters indicate differences between watering treatments across the two CO_2_ levels at *P* < 0.05 according to Duncan multiple range test. Vertical bars denote SE of the mean (*n* = 3–4)

Plant growth was increased significantly by applying water under both ambient and elevated CO_2_ (Fig. 2a). Elevated CO_2_ increased plant biomass by 21.6 %, 30.6 %, 32.3 %, 49.7 %, 52.1 %, 18.3 %, and 13.2 % in the W_−60_, W_−30_, W_−15_, W_0_, W_15_, W_30_, W_60_ watering treatments, respectively, indicating that the relationship between stimulation by CO_2_ enrichment and water status was a well-fitted quadratic function with higher points under moderate water change but declining under both water deficit and well-watered conditions. According to GLM ANOVA, CO_2_ concentration and watering treatment alone each significantly affected plant growth (*P* < 0.01, Additional file 3: Table S2). Plant individual leaf area significantly decreased with water deficit, whereas CO_2_ had no significant or systematic effect in GLM ANOVA (Fig. 2b; Additional file 3: Table S2).

From W_−60_ to W_0_, an increase in LAR with improving water status was observed under the ambient CO_2_ level; however, this increase trend seemed to be partly offset by elevated CO_2_ (Fig. 2c,). LARMR had a similar response to the water status gradient, and CO_2_ enrichment-induced attenuation of the response to water status change was also observed (Fig. 2d). Both watering and CO_2_ concentration had significant effects on these two parameters (*P* < 0.05, Additional file 3: Table S2).

**Fig. 3:**
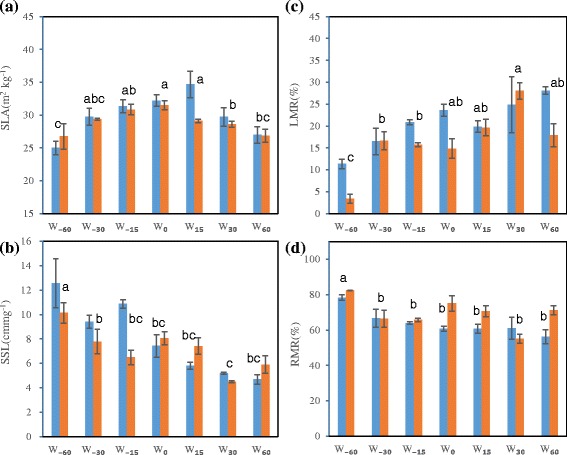
Effects of elevated CO_2_ on plant organ structure traits (**a**, SLA, specific leaf area; **b**, SSL, specific stem length) and biomass allocation (**c**, LMR, leaf mass ratio; **d**, RMR, root mass ratio) with water status gradient. For watering treatment and bar details, see Fig. 2

Watering had a significant effect on SLA, with a maximum under moderate water status and a reduction under water deficit or increased watering (Fig. 3a). SSL was also significantly affected by watering treatment, decreasing linearly with increased water (Fig. 3b). However, ANOVA on SLA and SSL suggested elevated CO_2_ and the interaction of watering and CO_2_ had no significant effect (Additional file 3: Table S2). LMR increased, while RMR decreased, with increasing water; in contrast, elevated CO_2_ seemed to reduce LMR and increase RMR in most cases except under W_30_ treatment (Fig. 3c, d). LMR and RMR were both significantly affected by watering, and LMR was also significantly affected by elevated CO_2_ (Additional file 3: Table S2).

### Photosynthetic capacity, stomatal conductance, and intrinsic WUE

**Fig. 4:**
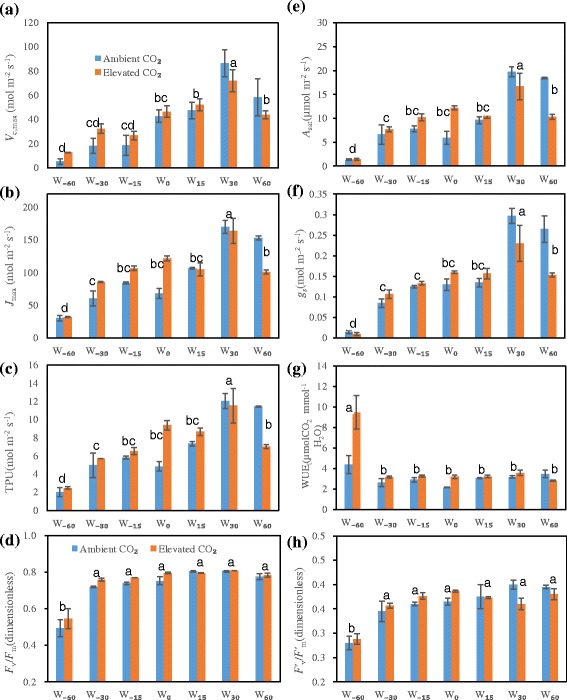
Effects of elevated CO_2_ on photosynthetic capacity (**a**, *V*_c,max_; **b**, *J*_max_; **c**, TPU; **e**, *A*_sat_), stomatal conductance (**f**, *g*_s_), intrinsic water use efficiency (**g**, WUE_i_), maximal photochemistry efficiency (**d**, *F*_v_/*F*_m_) and photochemical efficiency of open reaction centers of PSII (**h**, *F'*_v_/*F'*_m_) with water status gradient. *V*_c,max_, maximum rate of carboxylation; *J*_max_, maximum rate of electron transport, TPU, rate of thiose phosphate utilization; *A*_sat_, light-saturated maximum photosynthetic rate. For watering treatment and bar details, see Fig. 2

Watering had significant effects on the photosynthetic capacity parameters (*V*_c,max_, *J*_max_, TPU, *A*_sat_) with a maximum under W_30_ treatment, above or below which the values declined (Fig. 4). With elevated CO_2_, the photosynthetic capacity showed an increasing trend under relative water deficit, but a decrease under water surplus conditions (W_30_ and W_60_) (Fig. 4a–c, e). Stomatal conductance (*g*_s_) increased with increasing water, but showed a decreasing trend under W_60_, and was stimulated by elevated CO_2_, except under extreme drought and excess water conditions (W_30_ and W_60_) (Fig. 4f). WUE_i_ was increased only under extreme drought with ambient CO_2_, but was generally elevated under the high CO_2_ concentration except in the W_60_ treatment (Fig. 4g). Watering had significant effects on *V*_c,max_, *J*_max_, TPU, *A*_sat_, and *g*_s_, but no significant effect on WUE_i_ (Additional file 3: Table S2).

The two chlorophyll *a* fluorescence parameters—*F*_v_/*F*_m_ and *F'*_v_/*F'*_m_—were only significantly decreased by severe water deficit. Elevated CO_2_ did not have a significant effect on either parameter, except a slight stimulation under relative water deficit. *F*_v_/*F*_m_ was unchanged but *F'*_v_/*F'*_m_ was decreased by elevated CO_2_ under relatively sufficient water conditions (Fig. 4d, h). Watering alone had significant effects on *F*_v_/*F*_m_ and *F'*_v_/*F'*_m_, while CO_2_ concentration and the interaction of CO_2_ and water had no effect (Additional file 3: Table S2).

### Mesophyll cell ultrastructure

No significant changes in mesophyll cell length were observed under changes in the two treatment factors (Additional file 4: Table S3). Cell width increased with elevated CO_2_, but decreased with reducing water except in the W_30_ and W_60_ treatments. There was a linear increase in cell area with increasing water under ambient CO_2_; under elevated CO_2_, cell area was often increased with improving water status, but decreased with excess water. Cell wall thickness (CWT) was decreased only under extreme drought (W_−60_), but was drastically increased by CO_2_ enrichment by 22.2 % across all watering treatments. The chloroplast number per cell was decreased by extreme drought, but markedly increased by CO_2_ enrichment in all watering treatments. Although the three chloroplast size parameters (length, width, and profile area) showed no systematic responses to the water status gradient, they were decreased under elevated CO_2_ in plants subjected to extreme and severe drought (W_−60_ and W_−30_) and increased in the excess water treatments (W_30_ and W_60_). The number of grana thylakoid membranes (TMN) was unaffected by watering treatment, but was decreased by elevated CO_2_ by an average of 22.9 % across all watering treatments. Water deficit and relative water surplus caused declines in the starch grain number per chloroplast profile (SGN), and no intact starch grains were found at W_−60_ with ambient CO_2_. Elevated CO_2_ led to decreases in SGN in the W_−15_ and W_−30_ treatments, but increases in the other water treatments, indicating that the effect of elevated CO_2_ strongly depended on water status. The plastoglobuli number per chloroplast (PGN) tended to decrease under ample watering at ambient CO_2_; elevated CO_2_ seemed to decrease PGN under severe drought (W_−30_ and W_−60_), but increase it under excess water treatments. Based on ANOVA, CO_2_ concentration, watering, and their interaction all significantly affected CWT and PGN. Elevated CO_2_ and watering both had significant effects on the chloroplast number, but their interaction did not. Cell width, cell area, and SGN was significantly affected only by watering, and TMN only by elevated CO_2_. Finally, watering, and its interaction with CO_2_ concentration significantly affected the chloroplast length (Additional file 4: Table S3).

**Fig. 5:**
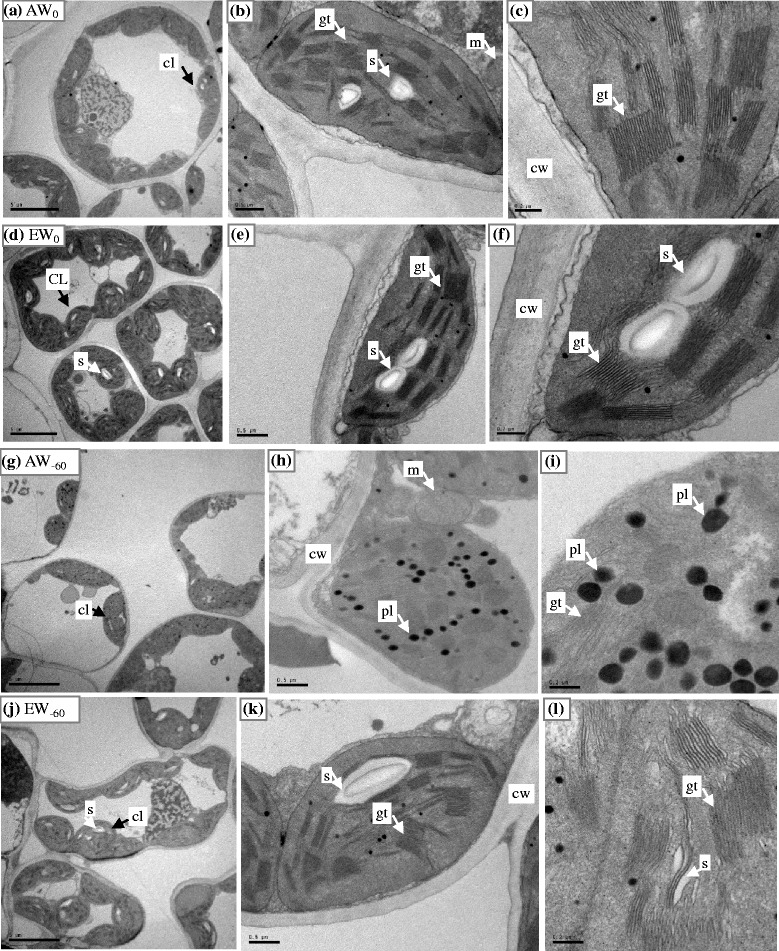
Transmission electron micrograph (TEM) of mesophyll cell in *Agropyron cristatum* leaves under control watering (W_0_, **a**-**f**) and severe water deficit (W_−60_, **g**-**l**) with ambient (**a**-**c**, **g**-**i**) and elevated CO_2_ (**d**-**f**, **j**-**l**) for whole mesophyll cell (**a**, **d**, **g**, **j**), whole chloroplast (**b**, **e**, **h**, **k**), and granum thylakoids (**c**, **f**, **i**, **l**). cl, chloroplast; cw, cell wall; gt, granum thylakoids; m, mitochondria; pl, plastoglobuli; s, starch grains. Bars, 5 μm (**a**, **d**, **g**, **j**), 0.5 μm (**b**, **e**, **h**, **k**), and 0.2 μm (**c**, **f**, **i**, **l**)

Furthermore, as directly seen from the transmission electron micrographs of mesophyll cells at different magnification scales (Fig. 5), the cells tended to become more circular under normal watering conditions and produced more chloroplasts under elevated CO_2_ (Fig. 5a, d). The starch grains in chloroplasts were more numerous and larger under elevated CO_2_ than under ambient CO_2_, and less grana thylakoid membranes were observed at the high CO_2_ level (Fig. 5b, c, e, f). An abnormally swollen grana thylakoid possibly extruded by the greater starch grains was also observed (Fig. 5f). A cell wall with distinct layers appeared (Fig. 5c, f). In plants exposed to extremely severe water deficit (Fig. 5g–l), very few chloroplasts were observed (Fig. 5g), the chloroplast envelope seemed to be broken, most of the chloroplast grana were swollen, grana thylakoids were unclear and appeared to have disintegrated, the cell wall was abnormal with uneven layers, and a number of large plastoglobuli were observed (Fig. 5h, i). However, under severe drought accompanied by elevated CO_2_, partial recovery seemed to have occurred, i.e., the damage was partly alleviated (Fig. 5j–l).

### Total water use and biomass water use efficiency

**Fig. 6:**
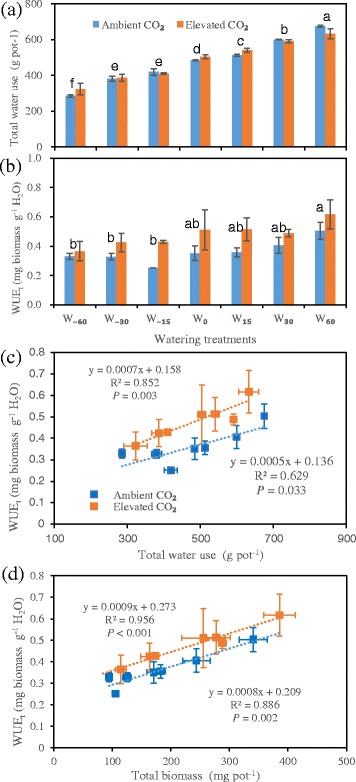
Effects of elevated CO_2_ on total water use (**a**), total biomass water use efficiency (**b**, WUE_t_) with water status gradient; and WUE_t_ relationships with total water use (**c**), and total biomass (**d**). In (**a**) and (**b**) panels, for watering treatment and bar details, see Fig. 2

Total water use, i.e., the actual evapotranspiration amount, significantly and linearly increased with increasing irrigation, from 304.6 to 654.5 g pot^−1^, a total increase of 114.9 % (Fig. 6a). However, elevated CO_2_ did not affect the total water use during the experimental period. WUE_t_ tended to increase with increasing water, particularly at the high CO_2_ concentration (Fig. 6b). WUE_t_ showed significant and strong relationships with total water use (Fig. 6c) and total biomass (Fig. 6d), particularly at the higher CO_2_ level, indicating that WUE_t_ is often higher in plants with a faster growth rate, even greater water consumption and under an increased CO_2_ concentration.

## Discussion

Our experiment on the effects of elevated CO_2_ on plants under various watering regimes showed that plant growth and photosynthetic capacity were stimulated by elevated CO_2_ under moderate water changes relative to normal precipitation; however, over-watering or extreme water deficit diminished or even eliminated this stimulation. The damage from severe drought, i.e., chloroplast and grana thylakoid damage, was partially ameliorated under the high CO_2_ level. This mostly confirmed our first hypothesis. This response to water status gradient at a high CO_2_ concentration was reflected by combined changes in plant architecture, biomass allocation, stomatal behavior, CO_2_ assimilation, PSII photochemical process, cell organelle structure, and water use efficiency (WUE), demonstrating highly synergistic changes at multiple scales. This coordinated response pattern may partly support our second hypothesis. Our results provide a deeper insight into the effects of varying water status on the response to CO_2_ enrichment, from cell ultrastructure to in vivo photosynthetic physiology and whole plant growth, highlighting that various aspects of the comprehensive responses of the dominant species need to be considered when assessing and projecting terrestrial ecosystem responses to climatic change, particularly in arid regions.

### Stomatal conductance

A reduction in *g*_s_ under enhanced CO_2_ can improve plant water status, thereby ameliorating the adverse effects of soil water deficiency on plant growth and physiological activity [8, 58]. As reported by Easlon et al. [59], a low *g*_s_ coupled with high photosynthetic capacity in *A. thaliana* plants growing under elevated CO_2_ might result from more conservative N investment in the photosynthetic apparatus. In the present experiment, the marked *g*_s_ decline due to water deficit seemed to be alleviated by elevated CO_2_, implying that increased CO_2_ has a protective role in drought-stressed leaves (Fig. 4f). In *Liquidambar styraciflua* plants, however, severe drought can lead to excessive stomatal closure and accelerated leaf senescence that may offset the benefits of CO_2_ enrichment for saving water [60]. The lack of benefit from increased CO_2_ to drought-stricken plants may be attributable to a lack of stomatal regulation [61]. Thus, the effect of elevated CO_2_ on the stomatal response to water change may depend on the species and extent of water deficit [58], but this needs to be explored further.

A marked decline in *g*_s_ under severe water deficit in close parallel with photosynthetic capacity may reflect the coexistence of stomatal and mesophyll limitations on photosynthesis (Fig. 4), in agreement with Flexas and Medrano [62]. Their results indicated that non-stomatal limitations including decreased photochemistry and Rubisco activity corresponded with decreases in *g*_s_, particularly below 100 mmol m^−2^ s^−1^. Photosynthetic mesophyll limitations, such as decreases in both photosynthetic activity and cell size, may play a major role in photosynthetic depression [63], which is almost in agreement with the present results. A recent report showed that *Ramonda nathaliae* plants with smaller stomata had higher resistance to drought than *R. serbica* [64], in line with the reductions in mesophyll cell size and *g*_s_ under severe drought in the present experiment (Additional file 4: Table S3). Thus, an associated change in cell size and stomatal behavior might confer a highly adaptive response to climate change.

### In vivo physiological capacity

Photosynthetic capacity parameters such as *A*_sat_, *V*_cmax_*J*_max_, and TPU are inhibited under extreme and severe drought, but elevated CO_2_ can partly alleviate this inhibition [4, 24, 26, 65, 66]. However, in the present results, although elevated CO_2_ increased the photosynthetic capacity under severe and moderate drought and control treatments, it had no significant effect, or caused a decrease, under surplus watering conditions (Fig. 4). As reported previously, the photosynthetic capacity was decreased under elevated CO_2_ in *Eucalyptus* seedlings grown under well-watered but not water-stressed conditions [46]. Thus, the positive effects of elevated CO_2_ on photosynthesis may favor plants exposed to a moderate range of water statuses—from severe to moderate water deficit stress.

The effect of elevated CO_2_ on in vivo chlorophyll *a* fluorescence is also uncertain. For example, the maximal photochemical efficiency of PSII (*F*_v_/*F*_m_) was increased by elevated CO_2_ in poplar seedlings [67]. However, chlorophyll fluorescence showed no changes under elevated CO_2_ in Scots pine needles [68] and some grasses [26]. Roden and Ball [46] showed that elevated CO_2_ led to a reduction of *F*_v_/*F*_m_ in amply watered *Eucalyptus* seedlings, but no effect was found under drought. No significant differences in *F*_v_/*F*_m_ were found in control and water-deprived *Phaseolus vulgaris* plants, although plant fresh weight decreased approximately 30 % in water-stressed conditions [69], suggesting that other metabolic processes related to growth, rather than PSII photochemical activity, might play a critical role in the response to water deficit stress. Our findings indicated that CO_2_ enrichment had no effect on *F*_v_/*F*_m_, and even inhibited the photochemical efficiency of open reaction centers of PSII (*F*′_v_/*F*′_m_) in relatively well-watered plants, but had a stimulatory effect under relative water deficit (Fig. 4h). Moreover, we found that accelerated accumulation in starch grains might damage the chloroplast structure under well-watered conditions, which might partly explain the depression of PSII activity.

In Rakić et al. [64], a reduction in *F*_v_/*F*_m_ occurred only in severely drought-stressed resurrection plants, consistent with our results in which a dramatic decline in *F*_v_/*F*_m_ and *F*′_v_/*F*′_m_ appeared only under extreme water deficit. Therefore, a drastic decline occurs only in plants subjected to extreme environmental stress, suggesting that these two parameters might not be good indicators of moderate water status changes. Elevated CO_2_ might not affect or might decrease these parameters, possibly because of decreased leaf thickness (greater SLA) or down-regulation of photosynthetic potential [26].

### Organelle structure changes

The chloroplast, a compulsory light-harvesting organelle, can be easily and seriously affected by elevated CO_2_ [70, 71]. Many previous studies have shown that elevated CO_2_ could increase the number of chloroplasts in mesophyll cells [47, 70, 72, 73], which was also confirmed by the current study (Additional file 4: Table S3, Fig. 5). However, the mechanism by which elevated CO_2_ positively regulates chloroplast numbers still remains unclear [47, 72]. Some reports have suggested it results from stimulation of chloroplast biogenesis processes [47, 74]. Additionally, there is other evidence for this abnormal change induced by increased CO_2_: a drastic increase in the amount of chloroplast stroma thylakoid membranes has been found relative to those in lower CO_2_ levels [70]. Damage to chloroplast ultrastructure can also occur under elevated CO_2_, partly as a result of increased starch grain size and numbers through accelerated starch accumulation in chloroplasts [47]. Enhanced accumulation of starch grains within chloroplasts by elevated CO_2_ can induce distortion of grana thylakoids, with plants exposed to a high CO_2_ level often having a low *A*_net_ [71]. In our current results, the increase in photosynthetic capacity was in agreement with the more numerous and larger starch grains in the chloroplasts of well-watered plants under the high CO_2_ level. It could be reasoned that starch accumulation might not be enough to limit the increase in photosynthesis induced by CO_2_ enrichment. However, this phenomenon would disappear under water deficit stress. Moreover, extremely severe water deficit can damage mesophyll cells, resulting in abnormal and disorganized cell organelles including chloroplasts and their grana [75], which was confirmed by the current experiment. However, this damage was partly ameliorated by elevated CO_2_, implying that plants have a strong dependence on the combination of CO_2_ concentration and water status.

### Plant structural traits and associations with physiological activities

Interestingly, LARMR increased with increasing water supply but decreased at the high CO_2_ level (Fig. 2d), reflecting different effects on the biomass investment balance between the light trapping organs and resource element-deriving organs from the two climatic factors [49, 76]. This indicates that elevated CO_2_ might negate the enhanced investment in light-intercepting organs under an over-watering regime in this species, in line with our earlier study [26]. PCA can not only unveil the extent and directions of correlations among plant structural and functional traits, but also distinguish the effects of treatment factors or their combinations from the projections, highlighting the importance of this useful analysis tool [23, 26, 57]. Here, for example, RMR and SMR had opposite distributions in the PC loadings (Fig. 1a), possibly reflecting the carbon allocation trade-off between root and stem organs [27]. Moreover, we found positive close relationships between plant structural traits and functional traits such as photosynthetic activities and *g*_s_; they were all positively related with PC1, which might highlight their coordinated changes under different climatic change factors (Fig. 1a). Close associations between morphological/structural and functional traits have been widely reported, depending on the species and environment [26, 77–79]. Our results again highlight that multiple variables at different scales might together play a critical role in the adaptive response to global change by balancing or offsetting each other.

### Water use efficiency

Elevated CO_2_ can improve plant water status by reducing *g*_s_ and thereby increasing WUE to ameliorate the adverse effects on plant growth and physiological processes from stress factors alone and in combination [4, 8, 58]. Water status also mediated the effectiveness of rising CO_2_ by coupling the processes of gas exchange and leaf enlargement. Nevertheless, the pros and cons of acclimation to changes in water conditions might coexist in plant responses to elevated CO_2_; leaf area enlargement induced by CO_2_ might exaggerate water use, while decreased *g*_s_ would promote WUE_i_ [15, 58]. However, WUE_i_ might decline under severe drought in some relict plant species exposed to elevated CO_2_ [61]. In the present experiment, both WUE_i_ and WUE_t_ were increased by elevated CO_2_, implying that the promotion of *A*_net_ and plant biomass by elevated CO_2_ might play a dominant role.

In the same steppe, conflicting results can occur because of different data types. For example, field rain use efficiency (RUE) increased with increasing MAP across different vegetation types, but decreased across different years in a given site, particularly in drier areas [40, 80]. Decreased RUE with increasing precipitation can be due to low productivity or other resource limitations such as N deficit [80]. WUE_t_ and RMR increased significantly with decreasing precipitation, but decreased with elevated CO_2_ [27]. In the present experiment, however, we found that the increase of WUE_t_ with increases in both water use and plant biomass (Fig. 6), particularly at a high CO_2_ level, might explain the resource limitation to WUE; both a water use increase and CO_2_ enrichment, as increases in available resources, might promote WUE by stimulating photosynthetic capacity and plant growth. Furthermore, our results indicated that although WUE_t_ and WUE_i_ showed a similar response to elevated CO_2_, the former was more sensitive, implying that WUE_t_ might be a better indicator than WUE_i_ for assessing responses to climate change [81].

### Elevated CO_2_ mitigation of severe drought

Elevated CO_2_ can ameliorate the negative effects of environmental stresses including severe water deficit in many different plant functional types or species such as the desert shrubs *Caragana intermedia* and *Caragana microphylla* [26, 82], the C_3_ perennial grasses *L. chinensis* and *Stipa grandis* [26, 27], and the C_4_ grass species *Cleistogenes squarrosa* [26]. The current experiment confirmed this amelioration due to CO_2_ enrichment in a C_3_ perennial grass from the same steppe as earlier experiments [26, 27, 82]. However, this amelioration was not observed in other reports on species such as some relict species [61], *Populus deltoides* [83], *L. styraciflua* [60], and *Eucalyptus radiata* [81]. Moreover, CO_2_ enrichment had a negligible effect on the response of *E. radiata* seedlings to drought, and did not alleviate the deleterious effects of drought due to rising temperature [81]. Thus, high CO_2_ may protect against or exacerbate stress effects, depending on different plant functional types and species.

A previous report showed that the growth of a dominant perennial shrub in a Mojave Desert ecosystem was doubled by a 50 % increase in CO_2_ only in a drier year [13]. In the present study, although elevated CO_2_ stimulated plant growth and photosynthetic activity in water deficit-treated plants, it had an inhibitory effect on the amply watered plants. This again indicates that CO_2_ enrichment may be more beneficial in drought conditions, implying that elevated CO_2_ may eliminate drought-induced negative plant responses. Thus, the allocation response to rising CO_2_ may also depend on water status. However, in a recent report, elevated CO_2_ productivity did no significantly modify the effects from soil water status in mesic grassland, semi-arid grassland, and xeric shrubland [84]. Taken together, these results suggest the integrated effects of elevated CO_2_ and water status on plants may be highly species- and habitat-specific.

## Conclusions

Elevated CO_2_ can improve plant water status and therefore stimulate various physiological and ecological processes from the organelle to cell, organ and plant individual level. However, this stimulation is strongly dependent on water status. Elevated CO_2_ generally increased the growth and photosynthetic physiological parameters of *A. cristatum* such as *V*_cmax_, *A*_sat_, *J*_max_, g_s_, TPU, and *F*_v_/*F*_m_ at severe to moderate water status, but had no effect on or even decreased these parameters in over-watering conditions. This indicates that CO_2_ enrichment can often ameliorate deleterious drought effects under moderate water deficit, but not extreme drought or over-watering conditions, and that plant morphological and structural alterations, and carbon allocation may be involved in this adaptive regulation. Our results for a dominant species from a degraded steppe suggest that water status changes such as severe drought or over-watering events might fundamentally contribute to the effects of CO_2_ enrichment on key physiological activities, cell structure, plant growth and even survival in a future climatic context, even completely reversing the direction of the effect. Our results highlight that CO_2_ fertilization’s dependency on water status should be considered when projecting plant responses to climate change. These findings contribute to our understanding of plant responses to global climate change, and may be useful in vulnerable ecosystem management.

## Abbreviations

*A*_sat_, light-saturated maximum photosynthetic rate; *F′*_v_/*F′*_m_, photochemical efficiency of open reaction centers of PSII; *F*_v_/*F*_m_, maximal PSII photochemical efficiency; *g*_s_, stomatal conductance; *J*_max_, maximum rate of electron transport; LAR, leaf area and plant total mass ratio; LARMR, leaf area and root mass ratio; LMR, leaf mass ratio; PCA, principal component analysis; RGR, relative growth rate; RMR, root mass ratio; SLA, specific leaf area; SMR, stem mass ratio; SSL, specific stem length; TPU, rate of thiose phosphate utilization; *V*_c,max_, maximum rate of carboxylation; WUE_i_, intrinsic water use efficiency; WUE_t_, total biomass water use efficiency.

## Additional files

Additional file 1: Figure S1. Changes in soil relative water contents (SRWC) at ambient and elevated CO_2_ concentrations with a water status gradient during a given period. Measured at 17:00 every three days, the watering day, but before watering during a consecutive 10-day period. W_−60_, W_−30_, W_−15_, W_0_, W_15_, W_30_, and W_60_ represent −60 %, −30 %, −15 %, 0, 15 %, 30 %, and 60 % of watering relative to mean precipitation in the local site over 30 years. Vertical bars denote SE of the mean (*n* = 3–4). GLM ANOVA refers to Additional file 2: Table S1. (DOCX 51 kb) Additional file 2: Table S1. Tests of between-subjects effects of CO_2_ concentration and watering on soil relative water content (SRWC) from GLM ANOVA. Bold font for *P* values indicates significance at *P* < 0.05. (DOCX 17 kb) Additional file 3: Table S2. Tests of between-subjects effects of CO_2_ concentration and watering on plant functional traits, physiological activity parameters from GLM ANOVA. Bold font for *P* values indicates significance at *P* < 0.05. (DOCX 24 kb) Additional file 4: Table S3. Mesophyll cell ultrastructure in *Agropyron cristatum* grown under ambient and elevated CO_2_ concentrations with water status gradient. (DOCX 21 kb)
