# Supplementary material for: Elevated CO2 can modify the response to a water status gradient in a steppe grass: from cell organelles to photosynthetic capacity to plant growth
Source: BMC Plant Biol. 2016 Jul 12;16:157. doi: 10.1186/s12870-016-0846-9 (PMC4942890; doi:10.1186/s12870-016-0846-9)
Supplement: Additional file 1: Figure S1. — Changes in soil relative water contents (SRWC) at ambient and elevated CO2 concentrations with a water status gradient during a given period. Measured at 17:00 every three days, the watering day, but before watering during a consecutive 10-day period. W−60, W−30, W−15, W0, W15, W30, and W60 represent −60 %, −30 %, −15 %, 0, 15 %, 30 %, and 60 % of watering relative to mean precipitation in the local site over 30 years. Vertical bars denote SE of the mean (n = 3–4). GLM ANOVA refers to Additional file 2: Table S1. (DOCX 51 kb) [file 12870_2016_846_MOESM1_ESM.docx]

**Figure S1.** Changes in soil relative water contents (SRWC) at ambient and elevated CO_2_ concentrations with a water status gradient during a given period. Measured at 17:00 every three days, the watering day, but before watering during a consecutive 10-day period. W_-60_, W_-30_, W_-15_, W_0_, W_15_, W_30_, and W_60_ represent -60%, -30%, -15%, 0, 15%, 30%, and 60% of watering relative to mean precipitation in the local site over 30 years. Vertical bars denote SE of the mean (n= 3-4). GLM ANOVA refers to Table S1.
