# Supplementary material for: Elevated CO2 can modify the response to a water status gradient in a steppe grass: from cell organelles to photosynthetic capacity to plant growth
Source: BMC Plant Biol. 2016 Jul 12;16:157. doi: 10.1186/s12870-016-0846-9 (PMC4942890; doi:10.1186/s12870-016-0846-9)
Supplement: Additional file 2: Table S1. — Tests of between-subjects effects of CO2 concentration and watering on soil relative water content (SRWC) from GLM ANOVA. Bold font for P values indicates significance at P < 0.05. (DOCX 17 kb) [file 12870_2016_846_MOESM2_ESM.docx]

**Table S1.** Tests of between-subjects effects of CO_2_ concentration and watering on soil relative water content (SRWC) from GLM ANOVA. Bold font for *P* values indicates significance at *P* < 0.05.

| Source | Days after sowing | Type III Sum of Squares | df | Mean Square | F | *P* |
| --- | --- | --- | --- | --- | --- | --- |
| CO_2_ level | 54 | 134.884 | 1 | 134.884 | 23.857 | **< 0.001** |
|  | 55 | 260.854 | 1 | 260.854 | 15.696 | **0.001** |
|  | 56 | 149.369 | 1 | 149.369 | 13.184 | **0.001** |
|  | 57 | 87.617 | 1 | 87.617 | 2.261 | 0.145 |
|  | 58 | 362.371 | 1 | 362.371 | 12.588 | **0.002** |
|  | 59 | 147.54 | 1 | 147.54 | 12.915 | **0.001** |
|  | 60 | 99.041 | 1 | 99.041 | 9.02 | **0.006** |
|  | 61 | 217.388 | 1 | 217.388 | 15.284 | **0.001** |
|  | 62 | 137.497 | 1 | 137.497 | 1.871 | 0.184 |
|  | | | | | | |
| Watering | 54 | 805.387 | 6 | 134.231 | 23.741 | **< 0.001** |
|  | 55 | 4076.36 | 6 | 679.393 | 40.881 | **< 0.001** |
|  | 56 | 1839.39 | 6 | 306.565 | 27.058 | **< 0.001** |
|  | 57 | 1581.07 | 6 | 263.512 | 6.799 | **< 0.001** |
|  | 58 | 4739.78 | 6 | 789.962 | 27.441 | **< 0.001** |
|  | 59 | 2915.03 | 6 | 485.838 | 42.527 | **< 0.001** |
|  | 60 | 2213.84 | 6 | 368.973 | 33.603 | **< 0.001** |
|  | 61 | 8572.64 | 6 | 1428.77 | 100.453 | **< 0.001** |
|  | 62 | 8602.6 | 6 | 1433.77 | 19.51 | **< 0.001** |
|  | 54 | 164.413 | 6 | 27.402 | 4.847 | **0.002** |
| CO_2_ * Watering | | | | | | |
|  | 55 | 414.297 | 6 | 69.05 | 4.155 | **0.005** |
|  | 56 | 104.292 | 6 | 17.382 | 1.534 | 0.208 |
|  | 57 | 242.338 | 6 | 40.39 | 1.042 | 0.422 |
|  | 58 | 263.718 | 6 | 43.953 | 1.527 | 0.210 |
|  | 59 | 64.359 | 6 | 10.726 | 0.939 | 0.485 |
|  | 60 | 58.402 | 6 | 9.734 | 0.886 | 0.519 |
|  | 61 | 167.671 | 6 | 27.945 | 1.965 | 0.109 |
|  | 62 | 206.979 | 6 | 34.497 | 0.469 | 0.824 |
