# Supplementary material for: Elevated CO2 can modify the response to a water status gradient in a steppe grass: from cell organelles to photosynthetic capacity to plant growth
Source: BMC Plant Biol. 2016 Jul 12;16:157. doi: 10.1186/s12870-016-0846-9 (PMC4942890; doi:10.1186/s12870-016-0846-9)
Supplement: Additional file 3: Table S2. — Tests of between-subjects effects of CO2 concentration and watering on plant functional traits, physiological activity parameters from GLM ANOVA. Bold font for P values indicates significance at P < 0.05. (DOCX 24 kb) [file 12870_2016_846_MOESM3_ESM.docx]

**Table S2.** Tests of between-subjects effects of CO_2_ concentration and watering on plant functional traits, physiological activity parameters from GLM ANOVA. Bold font for *P* values indicates significance at *P* < 0.05.

| Source | Type III Sum of Squares | df | Mean Square | *F* | *P* |
| --- | --- | --- | --- | --- | --- |
| **Plant biomass** |  |  |  |  |  |
| CO_2_ level | 1802.127 | 1 | 1802.127 | 10.038 | **0.004** |
| Water | 16965.79 | 6 | 2827.632 | 15.751 | **<0.001** |
| CO_2_ level * Water | 417.104 | 6 | 69.517 | 0.387 | 0.881 |
| Error | 5026.703 | 28 | 179.525 |  |  |
| Total | 138656.1 | 42 |  |  |  |
| Corrected Total | 24489.28 | 41 |  |  |  |
|  |  |  |  |  |  |
| **Leaf area** |  |  |  |  |  |
| CO_2_ level | 0.004 | 1 | 0.004 | 0.002 | 0.968 |
| Water | 120.574 | 6 | 20.096 | 7.455 | **<0.001** |
| CO_2_ level * Water | 8.664 | 6 | 1.444 | 0.536 | 0.776 |
| Error | 75.474 | 28 | 2.695 |  |  |
| Total | 601.893 | 42 |  |  |  |
| Corrected Total | 208.24 | 41 |  |  |  |
|  |  |  |  |  |  |
| **LAR** |  |  |  |  |  |
| CO_2_ level | 17.803 | 1 | 17.803 | 6.524 | **0.016** |
| Water | 105.89 | 6 | 17.648 | 6.467 | **<0.001** |
| CO_2_ level * Water | 16.291 | 6 | 2.715 | 0.995 | 0.448 |
| Error | 76.411 | 28 | 2.729 |  |  |
| Total | 1510.929 | 42 |  |  |  |
| Corrected Total | 223.654 | 41 |  |  |  |
|  |  |  |  |  |  |
| **LARMAR** |  |  |  |  |  |
| CO_2_ level | 101.461 | 1 | 101.461 | 6.774 | **0.015** |
| Water | 321.729 | 6 | 53.621 | 3.58 | **0.010** |
| CO_2_ level * Water | 60.185 | 6 | 10.031 | 0.67 | 0.675 |
| Error | 404.401 | 27 | 14.978 |  |  |
| Total | 4064.514 | 41 |  |  |  |
| Corrected Total | 908.454 | 40 |  |  |  |
|  |  |  |  |  |  |
| **SLA** |  |  |  |  |  |
| CO_2_ level | 9.16 | 1 | 9.16 | 0.864 | 0.361 |
| Water | 190.221 | 6 | 31.704 | 2.989 | **0.023** |
| CO_2_ level * Water | 42.346 | 6 | 7.058 | 0.665 | 0.678 |
| Error | 286.381 | 27 | 10.607 |  |  |
| Total | 36471.71 | 41 |  |  |  |
| Corrected Total | 542.044 | 40 |  |  |  |

**Table S2** (continued)

| Source | Type III Sum of Squares | df | Mean Square | *F* | *P* |
| --- | --- | --- | --- | --- | --- |
| **SSL** |  |  |  |  |  |
| CO_2_ level | 6.629 | 1 | 6.629 | 0.958 | 0.336 |
| Water | 170.412 | 6 | 28.402 | 4.107 | **0.004** |
| CO_2_ level * Water | 40.262 | 6 | 6.71 | 0.97 | 0.463 |
| Error | 193.649 | 28 | 6.916 |  |  |
| Total | 2794.548 | 42 |  |  |  |
| Corrected Total | 407.619 | 41 |  |  |  |
|  |  |  |  |  |  |
| **LMR** |  |  |  |  |  |
| CO_2_ level | 175.236 | 1 | 175.236 | 5.276 | **0.029** |
| Water | 1204.777 | 6 | 200.796 | 6.046 | **<0.001** |
| CO_2_ level * Water | 228.255 | 6 | 38.043 | 1.145 | 0.363 |
| Error | 929.905 | 28 | 33.211 |  |  |
| Total | 17195.49 | 42 |  |  |  |
| Corrected Total | 2614.162 | 41 |  |  |  |
|  |  |  |  |  |  |
| **RMR** |  |  |  |  |  |
| CO_2_ level | 318.625 | 1 | 318.625 | 3.796 | 0.061 |
| Water | 1576.673 | 6 | 262.779 | 3.131 | **0.018** |
| CO_2_ level * Water | 521.324 | 6 | 86.887 | 1.035 | 0.424 |
| Error | 2349.978 | 28 | 83.928 |  |  |
| Total | 191866.6 | 42 |  |  |  |
| Corrected Total | 4894.516 | 41 |  |  |  |
|  |  |  |  |  |  |
| ***V*_c,max_** |  |  |  |  |  |
| CO_2_ level | 14.529 | 1 | 14.529 | 0.044 | 0.837 |
| Water | 17433.56 | 6 | 2905.593 | 8.716 | **<0.001** |
| CO_2_ level * Water | 993.566 | 6 | 165.594 | 0.497 | 0.804 |
| Error | 7333.763 | 22 | 333.353 |  |  |
| Total | 87997.32 | 36 |  |  |  |
| Corrected Total | 25449.62 | 35 |  |  |  |
|  |  |  |  |  |  |
| ***J*_max_** |  |  |  |  |  |
| CO_2_ level | 59.437 | 1 | 59.437 | 0.104 | 0.75 |
| Water | 53883.24 | 6 | 8980.54 | 15.668 | **<0.001** |
| CO_2_ level * Water | 7550.226 | 6 | 1258.371 | 2.195 | 0.082 |
| Error | 12609.51 | 22 | 573.16 |  |  |
| Total | 453901.5 | 36 |  |  |  |
| Corrected Total | 74190.96 | 35 |  |  |  |

**Table S2** (continued)

| Source | Type III Sum of Squares | df | Mean Square | *F* | *P* |
| --- | --- | --- | --- | --- | --- |
| **TPU** |  |  |  |  |  |
| CO_2_ level | 1.407 | 1 | 1.407 | 0.308 | 0.584 |
| Water | 287.956 | 6 | 47.993 | 10.522 | **<0.001** |
| CO_2_ level * Water | 50.67 | 6 | 8.445 | 1.851 | 0.135 |
| Error | 100.346 | 22 | 4.561 |  |  |
| Total | 2360.394 | 36 |  |  |  |
| Corrected Total | 436.343 | 35 |  |  |  |
|  |  |  |  |  |  |
| ***A*_sat_** |  |  |  |  |  |
| CO_2_ level | 0.023 | 1 | 0.023 | 0.002 | 0.962 |
| Water | 896.863 | 6 | 149.477 | 14.934 | **<0.001** |
| CO_2_ level * Water | 148.828 | 6 | 24.805 | 2.478 | 0.055 |
| Error | 220.201 | 22 | 10.009 |  |  |
| Total | 4976.768 | 36 |  |  |  |
| Corrected Total | 1245.318 | 35 |  |  |  |
|  |  |  |  |  |  |
| ***g*_s_** |  |  |  |  |  |
| CO_2_ level | 0.001 | 1 | 0.001 | 0.334 | 0.569 |
| Water | 0.202 | 6 | 0.034 | 14.368 | **<0.001** |
| CO_2_ level * Water | 0.027 | 6 | 0.004 | 1.902 | 0.126 |
| Error | 0.052 | 22 | 0.002 |  |  |
| Total | 1.024 | 36 |  |  |  |
| Corrected Total | 0.279 | 35 |  |  |  |
|  |  |  |  |  |  |
| **WUE_i_** |  |  |  |  |  |
| CO_2_ level | 8.418 | 1 | 8.418 | 0.928 | 0.346 |
| Water | 63.277 | 6 | 10.546 | 1.163 | 0.361 |
| CO_2_ level * Water | 25.332 | 6 | 4.222 | 0.466 | 0.826 |
| Error | 199.53 | 22 | 9.07 |  |  |
| Total | 808.23 | 36 |  |  |  |
| Corrected Total | 315.576 | 35 |  |  |  |
|  |  |  |  |  |  |
| ***F*_v_/*F*_m_** |  |  |  |  |  |
| CO_2_ level | 0.005 | 1 | 0.005 | 1.101 | 0.305 |
| Water | 0.345 | 6 | 0.057 | 11.804 | **<0.001** |
| CO_2_ level * Water | 0.004 | 6 | 0.001 | 0.144 | 0.989 |
| Error | 0.112 | 23 | 0.005 |  |  |
| Total | 20.421 | 37 |  |  |  |
| Corrected Total | 0.469 | 36 |  |  |  |

**Table S2** (continued)

| Source | Type III Sum of Squares | df | Mean Square | *F* | *P* |
| --- | --- | --- | --- | --- | --- |
| ***F'*_v_/*F'*_m_** |  |  |  |  |  |
| CO_2_ level | 0.004 | 1 | 0.004 | 0.916 | 0.349 |
| Water | 0.345 | 6 | 0.057 | 11.75 | **<0.001** |
| CO_2_ level * Water | 0.004 | 6 | 0.001 | 0.143 | 0.989 |
| Error | 0.113 | 23 | 0.005 |  |  |
| Total | 20.421 | 37 |  |  |  |
| Corrected Total | 0.469 | 36 |  |  |  |
| Corrected Total | 0.469 | 36 |  |  |  |
