# Supplementary material for: Elevated CO2 can modify the response to a water status gradient in a steppe grass: from cell organelles to photosynthetic capacity to plant growth
Source: BMC Plant Biol. 2016 Jul 12;16:157. doi: 10.1186/s12870-016-0846-9 (PMC4942890; doi:10.1186/s12870-016-0846-9)
Supplement: Additional file 4: Table S3. — Mesophyll cell ultrastructure in Agropyron cristatum grown under ambient and elevated CO2 concentrations with water status gradient. (DOCX 21 kb) [file 12870_2016_846_MOESM4_ESM.docx]

**Table S3.** Mesophyll cell ultrastructure in *Agropyron cristatum* grown under ambient and elevated CO_2_ concentrations with water status gradient.

| Water | Cell L(μm)* | Cell W(μm) | Cell A(μm^2^) | CWT(μm) | Chl. No. | Chl. L(μm) | Chl. W(μm) | | Chl. A(μm^2^) | TMN | SGN | PGN |
| --- | --- | --- | --- | --- | --- | --- | --- | --- | --- | --- | --- | --- |
| Ambient CO_2_ concentration | | | | | | | | | | | | |
| W₋₆₀ | 16.46±2.26 | 10.63±1.14bc | 143.57±29.34b | 0.18±0.02c | 5.0±0.5b | 4.22±0.20 | | 2.27±0.10 | 7.52±0.49 | 18.0±1.5 | 0.0±0.0b | 11.8±2.9 |
| W₋₃₀ | 18.71±2.25 | 10.23±0.91c | 152.97±25.10b | 0.20±0.02bc | 7.0±0.4ab | 4.46±0.42 | | 2.32±0.30 | 8.49±1.91 | 13.7±2.0 | 0.5±0.3ab | 12.6±1.1 |
| W₋₁₅ | 17.28±1.97 | 12.21±0.98abc | 169.34±29.31ab | 0.21±0.01bc | 8.0±0.5ab | 4.42±0.20 | | 2.16±0.11 | 7.45±0.36 | 14.3±0.5 | 0.7±0.2ab | 12.2±0.4 |
| W₀ | 19.52±1.07 | 12.29±0.49abc | 189.49±14.71ab | 0.29±0.01a | 8.5±0.3ab | 4.13±0.15 | | 2.11±0.12 | 6.93±0.63 | 21.5±3.9 | 0.9±0.2ab | 7.2±1.1 |
| W₁₅ | 21.89±1.23 | 13.14±0.87ab | 229.82±28.28a | 0.23±0.01ab | 8.8±0.5ab | 4.43±0.14 | | 2.31±0.21 | 8.12±1.00 | 16.8±1.1 | 1.0±0.4a | 9.8±0.9 |
| W₃₀ | 20.63±1.97 | 13.01±1.03ab | 208.79±21.60a | 0.24±0.01ab | 9.3±1.1a | 4.50±0.20 | | 1.90±0.12 | 6.73±0.59 | 16.5±1.2 | 0.7±0.3ab | 6.5±1.1 |
| W₆₀ | 20.68±0.62 | 14.88±0.73a | 242.94±17.75a | 0.23±0.01ab | 8.2±0.3ab | 3.98±0.24 | | 2.08±0.15 | 6.61±0.82 | 12.0±0.6 | 0.7±0.2ab | 6.0±0.6 |
| Elevated CO_2_ concentration | | | | | | | | | | | | |
| W₋₆₀ | 17.05±1.16 | 10.91±0.48c | 146.16±11.45c | 0.24±0.02b* | 8.2±0.5c* | 3.62±0.17b | | 2.07±0.14 | 5.94±0.62 | 14.0±2.3 | 0.2±0.2d | 9.8±0.6cd |
| W₋₃₀ | 19.16±1.18 | 12.30±0.53bc | 187.29±19.01ab | 0.28±0.02ab* | 9.7±0.6bc* | 3.47±0.08b | | 1.70±0.08 | 4.65±0.25 | 11.0±1.1 | 0.3±0.2cd | 11.0±0.9bc |
| W₋₁₅ | 21.43±0.97 | 13.27±0.95abc | 222.98±18.20a | 0.30±0.01a* | 9.2±0.6bc | 5.08±0.49a | | 2.70±0.20 | 6.78±1.40 | 13.0±0.6 | 0.6±0.2bcd | 14.4±1.0a* |
| W₀ | 20.02±1.73 | 14.25±0.52ab | 178.99±22.00ab | 0.30±0.02a | 11.4±0.2a* | 4.27±0.22ab | | 1.99±0.21 | 6.77±0.95 | 14.7±1.6 | 1.5±0.2a | 13.0±1.6ab* |
| W₁₅ | 18.66±0.99 | 14.97±1.02a | 222.15±25.44a | 0.29±0.02a* | 9.5±0.3bc | 4.35±0.20ab | | 2.51±0.14 | 8.62±0.76 | 15.3±0.9 | 1.2±0.2ab | 12.4±0.5abc* |
| W₃₀ | 18.44±0.55 | 13.36±0.58ab | 193.26±9.45ab | 0.27±0.01ab* | 10.0±0.4ab | 4.80±0.27a | | 2.40±0.10 | 9.14±0.79 | 12.4±0.8* | 1.0±0.2abc | 11.6±1.0abc* |
| W₆₀ | 19.38±1.88 | 12.53±0.90bc | 161.44±40.20ab | 0.30±0.01a* | 10.8±0.8ab* | 4.74±0.22a | | 3.39±1.26 | 12.21±4.17 | 11.3±1.2 | 0.8±0.3bcd | 7.8±0.4d |
| *P* values from GLM ANOVA between temperature and watering, and their interaction | | | | | | | | | | | | |
| CO_2_ | 0.851 | 0.471 | 0.780 | <0.001 | <0.001 | 0.829 | | 0.324 | 0.301 | 0.035 | 0.222 | <0.001 |
| Water | 0.300 | 0.001 | 0.040 | <0.001 | <0.001 | 0.014 | | 0.613 | 0.397 | 0.108 | 0.001 | <0.001 |
| CO_2_×Water | 0.249 | 0.098 | 0.152 | 0.012 | 0.097 | 0.015 | | 0.407 | 0.099 | 0.830 | 0.764 | 0.039 |

*The objective length and width indicate the longest and widest dimensions, respectively. Cell L, cell length; Cell W, cell width; Cell A, cell profile area; CWT, cell wall thickness; Chl. No., Chloroplasts number per cell; Chl. L, chloroplast length; Chl. A, chloroplast area; TMN, grana thylakoid membranes number; SGN, starch grain number per chloroplast profile; PGN, plastoglobuli number per chloroplast. W_-60_, W_-30_, W_-15_, W_0_, W_15_, W_30_, W_60_ denote -60%, -30%, -15%, 0, 15%, 30%, and 60% of watering relative to mean precipitation in the local site over 30 years. CO_2_ and water represent CO_2_ concentration and watering treatments, respectively. Different lower case letters indicate significant differences between watering treatments at the same CO_2_ level at *P* < 0.05 according to Duncan multiple range test, while * indicates significant differences between CO_2_ level within same watering treatments (n = 9-27).
